# Improving Obesity and Insulin Resistance by Targeting Skeletal Muscle MKP-1

**Published:** 2020

**Authors:** Anton M. Bennett, Ahmed Lawan

**Affiliations:** 1 Department of Pharmacology, Yale University School of Medicine, New Haven, Connecticut 06520, USA; 2 Comparative Medicine, Yale University School of Medicine, New Haven, Connecticut 06520, USA; 3 Program in Integrative Cell Signaling and Neurobiology of Metabolism, Yale University School of Medicine, New Haven, Connecticut 06520, USA; 4 Department of Biological Sciences, University of Alabama in Huntsville, Huntsville, Alabama 35899, USA

**Keywords:** MAP kinase, MAP kinase phosphatase, Obesity, Insulin resistance

## Abstract

Obesity has reached a global epidemic and it predisposes to the development of insulin resistance, type 2 diabetes and related metabolic diseases. Current interventions against obesity and/or type 2 diabetes such as calorie restriction, exercise, genetic manipulations or established pharmacological treatments have not been successful for many patients with obesity and/or type 2 diabetes. There is an urgent need for new strategies to treat insulin resistance, T2D and obesity. Increased activity of stress-responsive pathways has been linked to the pathogenesis of insulin resistance in obesity. In this commentary, we argue that chronic upregulation of MKP-1 in skeletal muscle is part of a stress response that contributes to the development of insulin resistance, T2D and obesity. Therefore, inhibition of MKP-1 in skeletal muscle is a potential strategy for the treatment of T2D and obesity. We highlight therapeutic strategies for potential targeting of MKP-1 in skeletal muscle for the treatment of metabolic diseases as well as other diseases of skeletal muscle.

## Introduction

Obesity is a major public health problem globally. It develops as a result of an imbalance between energy intake and expenditure, and predisposes to the development of multiple diseases including insulin resistance, type 2 diabetes (T2D), nonalcoholic fatty liver disease (NAFLD), cardiovascular disease and some types of cancers [1–4]. Skeletal muscle constitutes about 30–40% of total body mass in healthy humans. Skeletal muscle is the major tissue involved in the control of energy balance and glucose metabolism. The generation, maintenance, composition and repair of skeletal muscle is considered to be an important aspect of metabolic homeostasis [5,6]. Dysfunctional energy metabolism in skeletal muscle has been attributed to the development of insulin resistance, glucose intolerance, T2D and obesity [7,8]. Myokines, are muscle-derived secretory molecules that mediate crosstalk between skeletal muscle and other organs/systems including the liver, adipose tissue, bone, pancreas and cardiovascular system [9]. Thus, maintenance of healthy skeletal muscle impacts virtually all organ systems in the body. Disruption of skeletal muscle function leads to numerous types of diseases including muscular dystrophy, loss of muscle mass (atrophy), and muscular hypertrophy [10–12]. Skeletal muscle is the main source of animal protein for human consumption, and the growth and development of skeletal muscle directly impacts animal meat quality and quantity [13]. Exercise has been the main benefit used by space agencies in order to protect skeletal muscle health while in space for prolonged periods of time [14,15]. Because skeletal muscle plays such a major role in metabolism the benefits of exercise have been shown to promote the actions of insulin and improve overall metabolic indicators. Thus, targeting skeletal muscle in cases of correcting metabolic dysfunction such as T2D could be considered a rational strategy.

The signal transduction pathways that govern metabolic regulation in skeletal muscle are extremely complex. Here we will focus on the regulation of the mitogen-activated protein kinase (MAPK) pathway in skeletal muscle. The MAPKs are a family of serine/threonine kinases that have been shown to be directly involved in skeletal muscle metabolism. The MAPKs are activated by direct phosphorylation by their upstream MAPK kinases (MKKs). In contrast, the inactivation of the MAPKs is achieved by direct dephosphorylation on both regulatory threonine and tyrosine residues by the MAPK phosphatases (MKPs) (16). The MKPs are a sub-family of enzymes known as dual-specificity phosphatases (DUSPs) that belong to a larger superfamily known as protein tyrosine phosphatases (PTPs). The archetypal MKP, MKP-1 has been studied extensively in a variety of cellular and biological systems. MKP-1 is abundantly expressed in skeletal muscle and is a critical negative regulator of p38 MAPK, c-Jun NH2 terminal kinase (JNK) and to a lesser extent, ERK activities [12,17,18]. We previously showed that MKP-1 is an important regulator of MAPK-dependent regulation of lipid homeostasis, energy metabolism, and mitochondrial biogenesis [16,18]. Work from this group using skeletal muscle-specific deletion of MKP-1 uncovered an important role of MKP-1 in this tissue. We demonstrated a major contribution of skeletal muscle MKP-1 in the regulation of glucose metabolism and energy homeostasis [18]. One of the most interesting observations of this work was the observation that MKP-1 is increased in its level of expression in skeletal muscle of obese humans [18]. These results, along with others that will be discussed here suggested that MKP-1 forms part of an important stress response that leads to reduced energy expenditure in skeletal muscle thereby contributing to weight gain. A paradigm consistent with the idea that stress positively correlates with obesity. Furthermore, it was discovered that MKP-1 participates through MAPK in crosstalk mechanisms with other tissues such as the liver to promote abnormalities in glucose metabolism and hepatic steatosis associated with metabolic disease [18]. Although MKP-1 expression has not been well studied in human obesity there is one study that reported increased MKP-1 expression in adipose tissue and macrophages in obese humans [19]. Our data is the first study that reported increased expression of MKP-1 in skeletal muscle of obese humans with concomitant dephosphorylation of p38 MAPK [18]. These findings are of significance because it implies that targeting skeletal muscle MKP-1 could be beneficial to the treatment of insulin resistance, T2D and obesity.

## Role of MKP-1 in Biological Processes and Disease Development

### Contribution of skeletal muscle MKP-1 in obesity and insulin resistance

**Figure 1:**
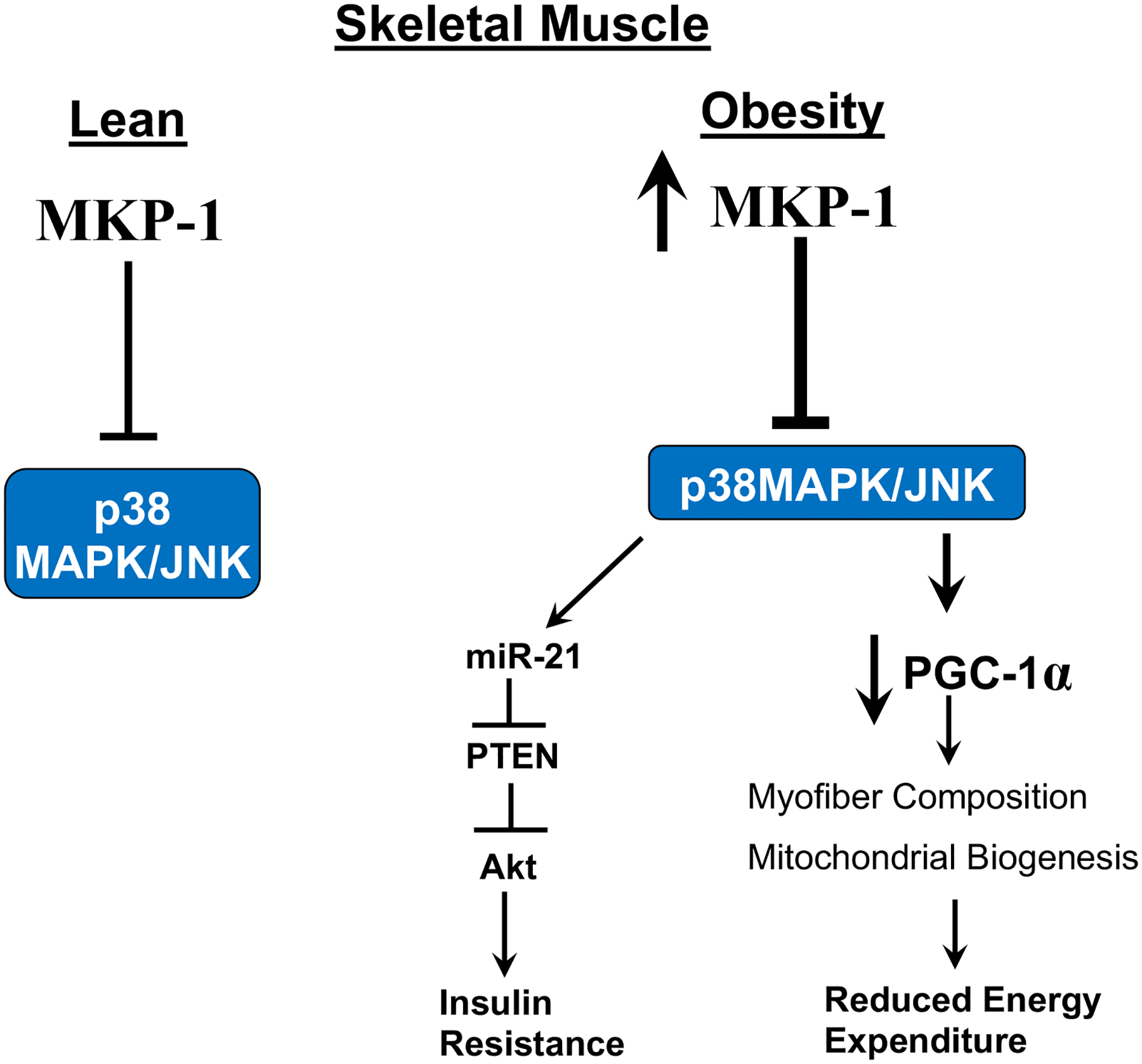
Model for MKP-1 regulation of MAPK pathway in obese skeletal muscle. MKP-1 regulate the activities of p38 MAPK/JNK pathway. In obesity, MKP-1 is upregulated, which inhibit p38 MAPK thereby impairing PGC1a activity leading to decreased Mitochondrial function and reduced energy expenditure. Overexpression of MKP-1 also leads to development of insulin resistance by controlling the activation of p38 MAPK/JNK through the miR-21/PTEN/Akt pathway.

MAPK signaling is necessary for the maintenance of skeletal muscle mass, regenerative myogenesis and muscle atrophy [12,20,21] and promotion of obesity-induced insulin resistance [22]. Overexpression of MKP-1 inhibits the expression and activity of PGC1a, a master regulator of mitochondrial biogenesis and energy expenditure, by impairing p38 MAPK-mediated PGC1a phosphorylation [17,18] (Figure 1). Further studies showed impaired muscle regeneration, reduced body weight, muscle mass, muscle cross-sectional area and exacerbated myopathy in MKP-1-deficient mice [12].

We showed that MKP-1 when deleted specifically in skeletal muscle relieves the inhibition of both JNK and p38 MAPK. Remarkably, instead of enhanced JNK/p38 MAPK activity driving obesity and insulin resistance as previous studies suggested [23], these mice are resistant to diet-induced obesity and are insulin sensitive [18]. Significantly, MKP-1 is upregulated in high-fat diet feeding in mice [17] and in obese in human subjects [18]. These data suggest that MKP-1 upregulation contributes to the development of obesity and insulin resistance by antagonizing the JNK/p38 MAPK signaling module. We found that enhanced p38 MAPK/JNK activities increased miR-21 expression in skeletal muscle lacking MKP-1, leading to reduced PTEN expression, thereby upregulating Akt activity. These results indicate that inhibition of MKP-1 in skeletal muscle could promote insulin sensitivity in part by MKP-1 mediated upregulation of Akt through a MAPK/miR-21/PTEN pathway (Figure 1). Further work is needed to substantiate the human data and definitively prove whether these findings in mice are recapitulated in humans. Here, using tissue-specific approaches the interpretation that the negative effects of skeletal muscle MKP-1 ultimately prevail in the development of obesity and insulin resistance has been established. This work shifts the entire idea that progression of obesity and insulin resistance as it pertains to JNK/p38 MAPK is simply a consequence of enhanced activity of these MAPKs. These findings raise the possibility that targeting MKP-1 in skeletal muscle may provide therapeutic potential for the treatment of obesity, insulin resistance and T2D.

### Skeletal muscle MKP-1 and its contribution in liver diseases

There is a growing body of literature that focuses on the role of skeletal muscle in the development of liver disease. Studies have shown impaired skeletal muscle function in NAFLD patients [24] and in chronic liver diseases including cirrhosis [25,26]. Whether the damage of skeletal muscle tissue is causal, a provoking factor or an effect of the chronic liver disease remains to be established. Also, the mechanism by which skeletal muscle affects liver diseases or whether alterations in skeletal muscle function contribute to the progression of liver disease is not known. We previously showed that mice lacking MKP-1 in the liver (MKP1-LKO) results in altered expression of hormones and cytokines along with resistance to hepatic steatosis [27]. Additionally, MKP1-LKO mice exhibit reduced mitochondrial function suggesting that deleting MKP-1 in the liver might inhibit skeletal muscle function possibly as a result of crosstalk that occurs between the liver and skeletal muscle tissue through a hepatic MKP-1-dependent pathway. Conversely, showed resistance to hepatic steatosis in mice lacking expression of MKP-1 in skeletal muscle. One potential mechanism for this crosstalk is the effect of myokines secreted from the skeletal muscle that mediate hepatic lipid metabolism. The exact molecular mechanism for this relationship has yet to be investigated.

### Skeletal muscle MKP-1 in mitochondrial function, sarcopenia and aging

Sarcopenia is defined by the gradual and extensive loss of skeletal muscle mass, strength and function [28]. It is a common complication observed in about 70% of liver cirrhosis patients [29,30] and it affects almost all elderly people [31]. The underlying mechanisms for the development of sarcopenia and the effective treatment for sarcopenia have not been discovered. Exercise is known to slow the progression of sarcopenia where it partially improves mitochondrial biogenesis and protein turnover. Sarcopenia is associated with increased obesity in the elderly. A decline in the proportion of type I myofibers, which are mitochondria rich, is observed in obese patients and the proportion of type I myofibers positively correlates with overall metabolic health [7–11,32]. Thus, the predominant view reflects the notion that the levels of oxidative myofiber composition negatively correlates with the development of metabolic syndrome. Indeed, this is consistent with the concept proposed here suggesting that skeletal muscle MKP-1 overexpression promotes insulin resistance and obesity by reducing oxidative myofiber composition (Figure 1).

The stress-responsive MAPKs control processes such as insulin signaling, glucose homeostasis, fatty acid metabolism and energy expenditure [33,34]. Mice with skeletal muscle-specific deletion of JNK1 are unaffected by diet-induced obesity but are insulin sensitive [35]. p38 MAPK has been shown to stimulate glucose uptake in muscle cells [36] and constitutive p38 MAPK activation in skeletal muscle promotes mitochondrial biogenesis [37]. Although it is realized that metabolic stressors such as inflammation and nutrient excess activate both p38 MAPK and JNK [38,39], the results of these studies reflect the individual actions of these MAPKs on metabolism. The studies described here on MKP-1 in skeletal muscle represent the integrated response of antagonizing these stress-responsive MAPKs on metabolism which has revealed the importance of MKP-1 as a crucial regulator in the progression of metabolic disease.

One of the main reasons for the actions of MKP-1/MAPK in skeletal muscle metabolism relates to the fact that this pathway regulates energy expenditure through controlling the composition of oxidative and glycolytic muscle fibers. By examining the composition of skeletal muscle myofibers we found that there was an increase in the proportion of slow oxidative myofibers and reduction in the proportion of fast glycolytic myofibers in skeletal muscle derived from mice lacking MKP-1 expression in this tissue [18]. Thus, our results imply that changes in myofiber type composition could contribute to the enhanced oxidative capacity and reduced glycolytic capacity of skeletal muscles in mice lacking skeletal muscle MKP-1. Mitochondrial oxidation, ATP synthesis, and myofiber type composition contribute to the control of skeletal muscle endurance [40,41]. Skeletal muscle-specific MKP-1-deficient mice were observed to exhibit significantly increased levels of endurance which, further supports the interpretation that the oxidative myofiber composition of these skeletal muscles is improved. These observations provide an explanation for the increased levels of mitochondrial efficiency and increased whole-body energy expenditure in skeletal muscle-specific MKP-1-deficient mice and thus, resistance to diet-induced obesity. These results suggest that skeletal muscle MKP-1 through a MAPK dependent pathway(s) modulates mitochondrial biogenesis and subsequently influences whole body energetics. Impaired mitochondrial biogenesis and protein turnover has been reported to promote the development of sarcopenia [30]. Therefore, overexpression of skeletal muscle MKP-1 would be anticipated to promote age- and diet-induced stresses on mitochondrial dysfunction and contribute to the progression of sarcopenia.

## MKP-1 in Cardioprotection

Heart disease and its associated complications including heart failure, atrial fibrillation and myocardial ischemia and infarction are leading causes of death and disability globally [42,43]. The molecular basis for the development and progression of cardiac diseases are not completely clear. Reperfusion is critical in rescuing the ischemic myocardium from infarction and cardiomyocyte death [43,44]. Insulin protects cardiac myocytes [45,46] while oxidative stress [44] has been implicated in ischemia-reperfusion induced cardiomyocyte apoptosis [44]. In cardiac myocytes, insulin resistance affects the cytoprotective effects of insulin that is mediated by induction of MKP-1 expression [47]. In a rat model of ischemia-reperfusion, dexamethasone-induced cardiomyocyte protection has been shown to be mediated by upregulation of MKP-1 expression [48]. In contrast, MKP-1 down-regulation by parathyroid hormone-related peptide has been shown to be cardioprotective [51]. It has been demonstrated that cardiac myocytes derived from MKP-1 knockout mice were protected from oxidative injury [49]. These studies demonstrate that the cardioprotective effects of MKP-1 are controversial. However, inhibition of MKP-1 protects against oxidative stress-induced myocytes apoptosis and modulation of the activity of MKP-1 during myocardial ischemia-reperfusion might be beneficial for cardiac function.

## MKP-1 as a Potential Therapeutic Target

### Rationale

There is significant interest in the role and implication of phosphatases in metabolic diseases. The ability to selectively target these signaling pathways holds tremendous therapeutic potential for the treatment of obesity and insulin resistance. Many studies have shown that MKP-1 may have important functions in health and disease [18,50–52]. Some of the roles for MKP-1 in obesity, energy homeostasis, insulin resistance, hepatic steatosis, diabetes and cardioprotection have been uncovered [17,27,50,53]. In the following section we will discuss the potential for targeting of MKP-1 as a potential strategy for the treatment of insulin resistance, T2D and obesity.

### Inhibition of MKP-1 in muscle

Several DUSPs have been implicated in human diseases including cancer, neurological and muscle disorders, metabolic disorders, inflammatory and cardiovascular diseases [52]. To date a combination of structural, biochemical, and genetic data unequivocally supports the conclusion that DUSPs exhibit overwhelming specificity towards the MAPKs [54,55]. MKP-1 is the prototypical member of this family of enzymes. Despite the fact MKP-1 has been extensively studied compared with other members of this family of enzymes, only recently has the crystal structure of the human MKP-1 catalytic domain been reported [56]. The absence of information about the crystal structure of this enzyme has hindered development of inhibitors to target MKP-1. Inhibitors of MKP-1 have been identified through the efforts of high-throughput screens [57]. The fact that the PTP domain of MKPs are all highly similar presents a challenge towards the development of potent and specific MKP-1 inhibitors. Since the crystal structure of MKP-1 has been solved, structure-based drug design is now possible.

Considering the contribution of MKP-1 in metabolic homeostasis, the ability to selectively target MKP-1 could be of great therapeutic potential for the treatment of metabolic diseases. In the last two decades kinases have appeared as a major class of druggable targets for the treatment of cancers and other diseases. Many drugs targeted against protein-tyrosine kinases (PTKs) [58] have had a significant impact on the treatment of various cancers [59]. However, many challenges exist with drugs targeted against PTKs for example, certain cancers treated with PTK inhibitors succumb to either intrinsic or acquired resistance to such treatments. Therefore, other targets and approaches are needed. Investigators both in industry and academia are working to find small molecule therapeutics targeting PTPs. However, these efforts to generate small molecules targeting the active site of PTPs have encountered challenges because of the polar nature of the active site [60,61]. New approaches such as allosteric inhibitors are being developed to target PTPs [58]. One such effort is in the development of allosteric inhibitors for PTP1B for the treatment of diabetes and obesity [62]. This strategy avoids formation of the active conformation of the enzyme by obstructing mobility of the catalytic center. Similarly, small molecule allosteric inhibitors of MKP-1 would be highly attractive target for therapeutic intervention in metabolic diseases including, insulin resistance, T2D and obesity.

There are other MKP inhibitors that have been developed but are poor [57]. However, recent work on the development of MKP-5 inhibitors has opened the door to novel strategies of allosteric modulation of the MKPs, in general, that can potentially be applied to MKP-1 [63]. The first co-crystal structure of a small molecule inhibitor with MKP-5 revealed an unknown allosteric site that resides within the catalytic domain of MKP-5. This allosteric site is conserved in many other MKPs, including MKP-1. It was demonstrated that key residues within the MKP-5 allosteric site are critical for defining compound binding and mutation of these residues in MKP-5 to those found in MKP-1 abrogates the ability of the compound to inhibit MKP-5. The catalytic domains of the DUSPs are between 36 and 57% identical to that of MKP-5 suggesting that sufficient differences exist in which specificity can be achieved. These results pave the way for the development of a new class of MKP-specific allosteric inhibitors. Given the renewed interest in the development of allosteric PTP inhibitors and its emerging success in other PTPs such as SHP-2 and PTP-1B, the development of MKP-1 allosteric inhibitors is an exciting therapeutic strategy.

In unstressed conditions MKP-1 is expressed at relatively low levels. However, in obese states and type 2 diabetes, MKP-1 is upregulated. Skeletal muscle MKP-1 is overexpressed in obese humans [18]. Thus, MKP-1 would be a good target for therapeutic intervention only in those diseased tissues where its expression is aberrantly increased. It is important to perform a systematic survey of human tissue-specific MKP-1 gene expression and splicing to unravel new opportunities for therapeutic target identification and evaluation. Development of a metabolic disease-targeted tissue-specific promoter system would be a desirable tool. This approach is already in use in the treatment of certain types of cancers [64]. To target skeletal muscle MKP-1 to treat metabolic diseases, there is need to design a dual promoter technology in which a skeletal muscle-specific transcription system under the control of a human alpha skeletal actin promoter antisense-based therapeutics against MKP-1. Recent studies have demonstrated tissue-specific oligonucleotide delivery that utilizes both viral and nonviral delivery vectors [65]. Strategies such as blocking expression of MKP-1 in skeletal muscle with antisense oligonucleotides could be beneficial in improving insulin sensitivity and prevent the development of obesity. Using the antisense-based therapeutics against PTP1B has shown efficacy in clinical trials [66,67].

Considering the fact that studies on cardioprotective effects of MKP-1 [45,47–49] including stress-responsive MAPK [68] are controversial, more studies are needed to elucidate how modulation of MKP-1 could be beneficial in the treatment of cardiac diseases. Future work will require cardioprotective analysis of MKP-1 in tissue-specific mouse models.

The encouraging message here is that inhibition of MKP-1 would be expected to provide a positive therapeutic value in the area of treating myocardial injury resulting from ischemic-reperfusion insults.

### Challenges

One of the challenges towards inhibiting MKPs are concerns about toxicity and/or adverse side-effects because it means removing a widely expressed MAPK antagonist. However, this is unlikely to be a major cause of concern since whole body deletion of MKP-1 [17,50] or specifically MKP-1 deletion in skeletal muscle [18] does not result in overt effects in unstressed animals. Furthermore, specific inhibition of MKP-1 causes distinct upregulation of the nuclear pool of MAPKs. Major side effects due solely to increased nuclear MAPK activation are anticipated to be greatly lessened because of the restricted effects on the nuclear pool of MAPKs. Inhibition of MKP-1 pharmacologically in skeletal muscle, possibly through the development of drugs that can be targeted selectively to this tissue, will result in the activation of the nuclear pool of MAPKs causing upregulation of genes that promote energy expenditure without affecting the cytosolic pool of MAPKs that interfere with insulin sensitivity. Collectively, these mechanisms due to their spatiotemporally restricted effects, could afford highly favorable and tolerable long-term side-effects. The validity of these ideas will need to be rigorously tested in an assortment of mouse models of metabolic disease.

## Conclusion

After over a decade of study of the physiological function of MKP-1 in the regulation of metabolic homeostasis in mice and more recently in humans, these data collectively point to the notion that chronic upregulation of MKP-1 in skeletal muscle is part of a stress response mediated by p38 MAPK and JNK activities and this may play an important contributing role in the development of insulin resistance, T2D and obesity. Despite MKP-1 being a challenging, target there are potential strategies that if successfully executed could lead to inhibiting MKP-1 as a treatment of metabolic disease.
